# Insights from targeting transferrin receptors to develop vaccines for pathogens of humans and food production animals

**DOI:** 10.3389/fcimb.2022.1083090

**Published:** 2023-01-06

**Authors:** Nikolas F. Ewasechko, Somshukla Chaudhuri, Anthony B. Schryvers

**Affiliations:** ^1^ Department of Microbiology, Immunology and Infectious Diseases, Cumming School of Medicine, Calgary, AB, Canada; ^2^ Department of Production Animal Health, Faculty of Veterinary Medicine, University of Calgary, Calgary, AB, Canada

**Keywords:** transferrin, TBDT, evolution, vaccine, microbioal community, host specifity, iron acquisition systems

## Abstract

While developing vaccines targeting surface transferrin receptor proteins in Gram-negative pathogens of humans and food production animals, the common features derived from their evolutionary origins has provided us with insights on how improvements could be implemented in the various stages of research and vaccine development. These pathogens are adapted to live exclusively on the mucosal surfaces of the upper respiratory or genitourinary tract of their host and rely on their receptors to acquire iron from transferrin for survival, indicating that there likely are common mechanisms for delivering transferrin to the mucosal surfaces that should be explored. The modern-day receptors are derived from those present in bacteria that lived over 320 million years ago. The pathogens represent the most host adapted members of their bacterial lineages and may possess factors that enable them to have strong association with the mucosal epithelial cells, thus likely reside in a different niche than the commensal members of the bacterial lineage. The bacterial pathogens normally lead a commensal lifestyle which presents challenges for development of relevant infection models as most infection models either exclude the early stages of colonization or subsequent disease development, and the immune mechanisms at the mucosal surface that would prevent disease are not evident. Development of infection models emulating natural horizontal disease transmission are also lacking. Our aim is to share our insights from the study of pathogens of humans and food production animals with individuals involved in vaccine development, maintaining health or regulation of products in the human and animal health sectors.

## Introduction

**Figure 1:**
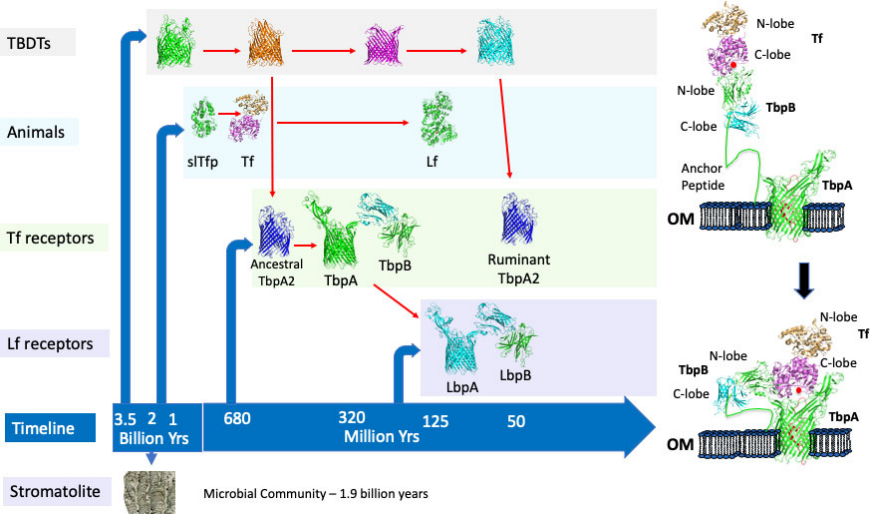
The evolution of iron acquisition systems in bacteria and multicellular animals. The timeline for appearance of microbial communities (stromatolite), proteins involved in acquisition of iron by Gram-negative bacteria (TBDTs, Tf receptors, Lf receptors) and in multicellular animals is illustrated in the left panel. Red arrows indicate evolutionary ancestors. The process for capture and iron removal from the two-component receptor is illustrated in the right panel.

The original isolation and identification of receptor proteins for the human iron-binding proteins, transferrin and lactoferrin in the human pathogens *Neisseria meningitidis* and *N. gonorrhoeae* (Schryvers and Morris, 1988a; Schryvers and Morris, 1988b) raised the question of whether this was a unique feature of the pathogenic *Neisseria* species or whether these type of receptors could be found in bacterial pathogens in other animal hosts. This search initially led to the identification of transferrin receptors (**Figure 1**, right panel) in the porcine pathogen *Actinobacillus pleuropneumoniae* (Gonzalez et al., 1990), the bovine pathogens *Pasteurella (Mannheimia) haemolytica* (Ogunnariwo and Schryvers, 1990) and *Haemophilus somnus (Histophilus somni)* (Ogunnariwo et al., 1990) and the poultry pathogen *Haemophilus (Avibacterium) paragallinarum* (Ogunnariwo and Schryvers, 1992). A more comprehensive search ultimately led to identifying transferrin and lactoferrin receptors from bacteria in three lineages (represented by the families Neisseriaceae, Moraxellaceae and Pasteurellaceae) in humans, pigs, horses and several ruminant species (Gray-Owen and Schryvers, 1996). Since these initial approaches relied on the ability to purchase or produce transferrin or lactoferrin and on acquiring collections of clinical isolates, it was not readily extended to a broader range of mammalian or vertebrate lineages. Nevertheless, this limited survey leads to the expectation that there will be bacteria that possess these receptors residing in the upper respiratory tract of virtually all mammalian and bird lineages and may even be present in terrestrial reptilian lineages.

The discovery of the transferrin receptors in *N. meningitidis* and the inference that they would be important both during invasive infection and for survival on the mucosal surface of the upper respiratory tract led to the pursuit of the receptor proteins for a vaccine against Group B meningococci (Danve et al., 1993). Preliminary results prompted the selection of transferrin binding protein 2 (Tbp2, now termed TbpB, **Figure 1**) as the vaccine antigen (Lissolo et al., 1995) and, although there was considerable sequence diversity, a vaccine comprised of 2 - 3 TbpBs was expected to provide comprehensive cross-protection (Rokbi et al., 1997). The observation that the transferrin receptor in *N. gonorrhoeae* was required for experimental infection of the human male urethra in strains naturally lacking lactoferrin receptors (Cornelissen et al., 1998) provided additional support for targeting the transferrin receptor in a vaccine. However, a decision to pursue other options than the TbpB-based vaccine led to the development of the commercial Bexsero and Trumenba vaccines against Group B meningococci by novel approaches.

Solving the structure of the TbpB from the porcine pathogen *A. pleuropneumoniae* (Moraes et al., 2009) and subsequent studies (Calmettes et al., 2011; Yang et al., 2011) demonstrated that this surface lipoprotein has a long anchoring peptide that would allow it to extend from the outer membrane to beyond the polysaccharide capsule to capture the iron-loaded form of transferrin (**Figure 1**, right panel) and deliver it to transferrin binding protein A (TbpA) through an interaction involving the N-terminal segment of the anchoring peptide. Subsequently, the structure of the human transferrin-TbpA complex was solved (Noinaj et al., 2012), providing insights into the process of iron removal and transport through the TbpA channel, using energy derived from an inner membrane-anchored energy transfer complex (TonBExbBExbD) (Postle, 1993) that is common to all the TonB-dependent transporters (TBDTs). The discovery of a novel single component receptor in bovine strains of *P. multocida* that bound to the N-lobe of transferrin (Ogunnariwo and Schryvers, 2001) (**Figure 1**, Ruminant TbpA2), contrasting the binding of the C-lobe of transferrin by the two component receptors present in most species, was surprising considering that the C-lobe is preferentially iron-loaded under normal physiological conditions. The only homologue that has been identified in other species is a TbpA2 receptor protein in strains of *H. somni* (Ekins et al., 2004) which may have been acquired from *P. multocida.* Since this receptor is only present in ruminants, and preferentially binds to the N-lobe, it could potentially reflect adaptation to unique physiological conditions in the oropharyngeal region of ruminants due to their adaptation for digesting grasses.

The availability of structural information provided the opportunity to design modified antigens derived from TbpB and TbpA with potential for improving their immunological properties. The first modified antigen that was developed was a site-directed mutant of a truncated TbpB protein from the porcine pathogen *Glaesserella (Haemophilus) parasuis* that provided complete protection from an intra-tracheal challenge with *G. parasuis* in colostrum-deprived piglets (Frandoloso et al., 2015). In contrast, the wild-type TbpB or a commercial vaccine preparation provided limited protection in this infection model. An intranasal challenge model was developed for *G. parasuis* to provide a more natural route of infection and the mutant TbpB was again shown to provide complete protection against infection (Frandoloso et al., 2020). Notably, the pigs were sampled for natural colonization prior to challenge and the colony counts were reduced or eliminated in pigs immunized by the intramuscular route by a standard vaccine formulation or by needle-free application of a novel microparticle preparation. Structural information was also used to design a novel hybrid antigen consisting of a modified C-lobe of *N. meningitidis* TbpB displaying a surface loop from the TbpA protein to overcome challenges for commercial production of a TbpA-based vaccine (Fegan et al., 2019). The hybrid antigen was able to induce antibodies that were equally capable as those produced against TbpA in binding to TbpA, inhibiting growth of an iron-starved TbpB-deficient mutant strain and inducing bactericidal antibodies.

## An evolutionary perspective

Gram-negative bacteria have been present for a substantial portion of the Earth’s history as there are fossils of microbial communities containing Cyanobacteria (stromatolites, **Figure 1**) that are nearly 2 billion years old and Cyanobacteria have been implicated in the gradual oxygenation of the Earth’s atmosphere and oceans from 2.7 to 1.7 billion years (Sosa Torres et al., 2015; Large et al., 2022). The primordial seas had substantial levels of ferrous iron and the increased oxygen levels converted ferrous to insoluble ferric ions and ultimately led to the generation of the ozone layer that eventually made life on land possible. The reduction in available iron in the oceans led to the production and secretion of iron-chelating molecules by members of the microbial community along with systems to capture and transport the iron complexes. The uptake of iron complexes is mediated by TBDTs that are present in Gram-negative bacteria that synthesize the iron-chelating molecules (including high affinity siderophores) as well as bacteria that are dependent upon siderophores produced by neighboring organisms (D'Onofrio et al., 2010).

The common features of iron binding proteins found in diverse animal lineages has led to the proposal that bilobed iron binding proteins such as transferrin were generated by a gene duplication event at least 670 million years ago and perhaps nearly a billion years ago (Lambert et al., 2005; Lambert, 2012) (**Figure 1**). Although somewhat speculative, the role of the single-lobed transferrin precursor (slTfp) was likely initially related to the efficient capture of iron from seawater. It is generally accepted that another gene duplication event in mammals led to the appearance of lactoferrin (Lambert et al., 2005), but the molecular evolution in this family of proteins is considerably more complex (Lambert, 2012).

The bacterial transferrin receptors are exquisitely specific for host Tf (Gray-Owen and Schryvers, 1993) and in an elegant study based on positive selection analysis the authors concluded that host specificity of receptors for primate transferrin had evolved over 40 million years of primate evolution (Barber and Elde, 2014). Since the host-specific bacterial transferrin receptors are present in bacteria that reside in the upper respiratory tract of mammals and birds, the implication is that they have existed for over 320 million years, when the Sauropsida and Synapsida lineages diverged (Ostan et al., 2021) (**Figure 1**, TbpA and TbpB). Unlike the host-specific two-component receptors in bacteria that colonize the different mammalian, avian and terrestrial reptilian lineages, which evolved from ancestral receptors, the single component TbpA2 only present in ruminants, likely evolved instead from an existing TBDT involved in iron transport (**Figure 1**) as it extracts iron from the N-lobe of Tf. This evolutionary process likely also occurred for development of an ancestral TbpA2 (**Figure 1**) that bound the C-lobe of Tf and was ultimately replaced by the more efficient two component receptor. The appearance of lactoferrin in the mammalian lineage due to a duplication of the transferrin gene (Lambert et al., 2005) likely resulted in both transferrin and lactoferrin being available on the mucosal surface so that the co-evolution of the bacterial receptor with this new transferrin would eventually lead to a lactoferrin receptor (LbpA and LbpB) specific for host lactoferrin (**Figure 1**).

The bacterial lineages that led to the Pasteurellaceae, Neisseriaceae and Moraxellaceae families likely arose early since there are bacteria in diverse ecological niches in these lineages. This raises the question why transferrin receptors have only been found to date in bacteria from the Pasteurellacea in avian hosts (Ogunnariwo and Schryvers, 1992) and why lactoferrin receptors did not appear in the Pasteurellacea.

## Do iron acquisition capabilities influence ‘biogeographical distribution’ of commensals and pathogens?

**Figure 2:**
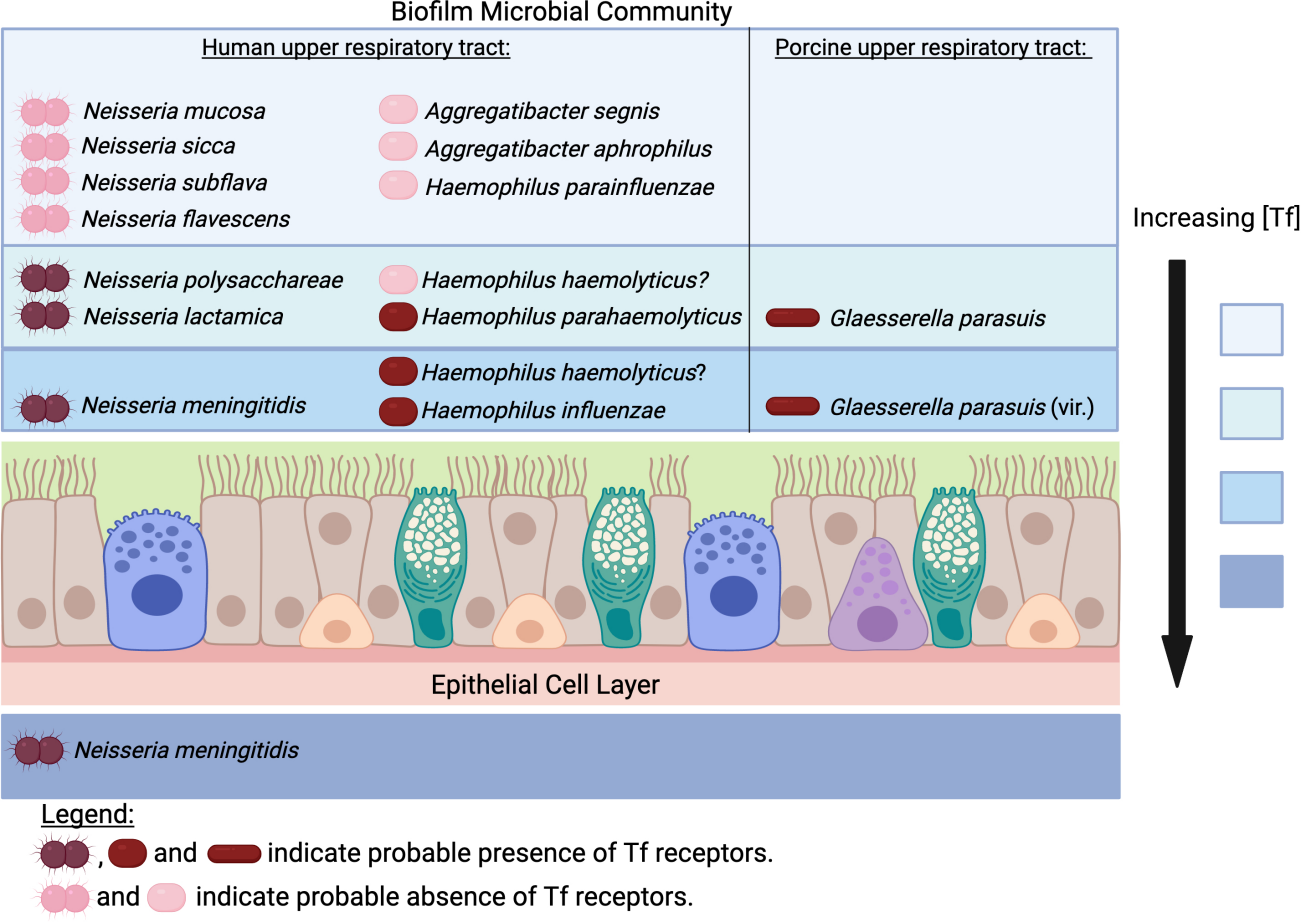
Does transferrin availability vary in the upper respiratory tract for pathogens and commensals? The concentration of transferrin (Tf) would be expected to be highest in the subepithelial space and decrease the further away from the epithelial cell layer the biofilm extends. In contrast, the concentration of iron from environmental sources would be highest at the air-liquid interface. The rationale for the proposed localization for *Neisseria* species is described in the text. The determination of the prevalence of receptors in *Neisseria* species has been described previously (Chan et al., 2018) and the same approach was used to evaluate the presence of receptors in the bacteria from the Pasteurellaceae. Access to Tf would not provide a tendency for *H. parainfluenzae, A. aphrophilus* and *A. segnis* to localize near the mucosal surface since they do not possess Tf receptors. *H. parahaemolyticus* strains possess Tf receptors whereas only some strains of *H. haemolyticus* do. However, the demonstrated competition between *H. haemolyticus* and *H. influenzae* (Atto et al., 2021) and similarity in adhesins (Singh et al., 2013) suggests that at least some strains of *H. haemolyticus* occupy the same niche. All strains of *Glaesserella parasuis* possess Tf receptors, with more virulent serovars possessing genes involved in binding to the mucosal surface. (Created with Biorender.com).

The important pathogens of humans and food production animals that possess transferrin receptors (+/- lactoferrin receptors) are from lineages that include related bacteria that may or may not contain transferrin receptors (+/- lactoferrin receptors) but rarely cause infection. These bacteria primarily reside in microbial communities on the nasal, nasopharyngeal or oral mucosal surfaces of their mammalian host and are transmitted by the respiratory route. A subset of these bacteria is sexually transmitted. The presence of transferrin on the mucosal surface is inferred by the presence of the receptor proteins, and probably the best information on the relative amounts of transferrin and lactoferrin on mucosal surfaces was obtained by measuring their presence in urine in a human gonococcal infection study (Anderson et al., 2003). Studies of microbial communities associated with infections suggested that 80% of the bacteria in microbial communities associated with infections are present within spatially structured microbial communities (Azimi et al., 2022). This raises the question of whether the biogeography of ‘normal’ microbiota is also spatially structured and to what extent this involves preferential localization in the vertical dimension (**Figure 2**). Regarding the bacterial lineages that possess transferrin and lactoferrin receptors that inhabit microbial communities on respiratory and oral mucosal surfaces, this question can be extended to how this relates to their relative abilities to acquire host and environmental sources of iron.

In the well-studied human-colonizing *Neisseria* species, the tendency to localize adjacent to the mucosal epithelial cells is associated with the Opa (opacity) proteins that are not present in commensal *Neisseria* species (Wanford et al., 2018). Studies with transgenic mice expressing human CEACAM1 demonstrated an increase in the extent and duration of Opa-dependent colonization, and imaging confirmed that the Opa-expressing bacteria in CEACAM1-humanized mice were tightly associated with the mucosal epithelial cells in contrast to control mice in which bacteria were visualized at different distances from the mucosal surfaces (Johswich et al., 2013). It is important to consider that the tight adherence by Opa proteins to mucosal epithelial cells can trigger transcytosis across the epithelial cell layer, which would also provide enhanced access to transferrin, but survival would rely on additional mechanisms unique to the pathogenic *Neisseria* (Gray-Owen and Blumberg, 2006) (**Figure 2**). The availability of transferrin on the mucosal surface will likely be greater closer to the epithelial cells, thus commensal species that usually possess transferrin receptors such as *N. lactamica, N. polysacchareae* and *N. cinerea* may tend to grow more effectively if located closer to the mucosal surface. The commensal species that are variable in the possession of transferrin receptors such as *N. sicca, N. mucosa, N. subflava* and *N. flavescens* (Chan et al., 2018) clearly are capable of surviving without accessing transferrin, thus likely can use alternate environmental iron sources, including iron complexed by siderophores available within the microbial community, and their distribution within the biofilm may reflect that (**Figure 2**). Recent genomic studies indicate that the phylogeny of human-colonizing *Neisseria* species is more complex with seven additional species (Diallo et al., 2019), which could provide a more comprehensive picture of the relationship between iron acquisition mechanisms and biogeographic distribution.

Applying similar logic to other bacterial lineages that are present in human and animal hosts might suggest a similar vertical distribution of ‘pathogens’ and ‘commensals’ (**Figure 2**), which may provide unique opportunities for probing this question with the spectrum of experimental tools and approaches that continue to evolve (Azimi et al., 2022). It may also be important to consider whether the ‘biogeography’ might impact the detection of the different bacterial species in the microbial community, especially when different sampling methods are used in different studies aimed at evaluating the relative prevalence of these organisms. This is particularly true in host species such as mice where either swab or lavage samples of the tissues tend to be implemented in different instances.

## Development of infection models

The options for developing infection models are clearly different for pathogens of humans and food production animals, but the relevance of extrapolating results from one host species to another is a common issue. The question of whether demonstration of efficacy of a vaccine directed against pigs (Frandoloso et al., 2015; Frandoloso et al., 2020) provides ‘proof of concept’ for potential success in developing vaccines against human pathogens to some extent is similar to the question whether mouse infection models can be used to predict the success of human or animal vaccines. We see these two approaches as complementary. More confidence in the relevance of each could be gained when mouse infection models are implemented alongside infection models in pigs or cattle and if the mouse models are able to predict relative efficacy of vaccine antigens or formulations.

One of the major limitations of mouse infection models is that they may not reflect host-specific interactions that are essential for disease pathogenesis. This can be overcome by using an appropriate mouse pathogen such as the case with *Citrobacter rodentium*, which has proven to be an invaluable model for *Escherichia coli* infection (Crepin et al., 2016). The isolation of *Neisseria musculi* from wild mice (Weyand et al., 2016; Ma et al., 2018) provided many insights into common features between the species present in mice and humans, but clearly does not serve as a fully relevant model for the pathogenic *Neisseria* in humans as it lacks both transferrin and lactoferrin receptors. It would be surprising if all the ancestral transferrin and lactoferrin receptors from three different bacterial lineages disappeared during the 70 million years of rodent evolution (Swanson et al., 2019), but it may require a more targeted strategy that uses samples other than oral swabs from wild mice and perhaps other rodent species to isolate the bacteria. It is also possible that it they will ultimately be discovered through the increasing availability of genomic sequences of bacteria in public databases.

The natural route of infection for most of the pathogens expressing Tf and Lf receptors likely involves a close association with the epithelial layer of the mucosal surface along the respiratory tract, and proliferation due to a ready source of iron on the mucosal surface or submucosal space after crossing the epithelial cell layer. However, most infection models involve directly administering a large dose of the bacteria to the lung or peritoneal cavity where bacterial-derived toxins may play a role in disease pathogenesis (Conlon et al., 1991; Tatum et al., 1998), so toxin neutralization may be required for full protection in this non-physiologic disease model. In our standard model for *N. meningitidis* infection, we grow the bacteria under iron-limited conditions to induce expression of the receptors and deplete their ferritin iron stores to reflect the physiological conditions *in vivo*, and then provide exogenous human Tf as a physiologically relevant iron source. We have considered using this as a surrogate infection model for targeting other iron acquisition proteins by expressing the foreign gene in the *tbp* or *lbp* locus. Recent studies have shown that it is possible to induce experimental Glässer’s disease in pigs with an optimal dose of *Glaesserella parasuis* bacteria administered by the intranasal route (Frandoloso et al., 2020), which can be prevented by a vaccine targeting a single surface antigen (TbpB), suggesting that optimizing the dose for intranasal challenge by other pathogens could be considered. The seeder pig challenge-exposure model provides the most natural route of infection (Lechtenberg et al., 1994), and modifications of the experimental design could potentially enhance the efficiency of disease transmission when applied to pigs or perhaps to other animal species.

## Limitations of bacterins and conjugate capsular vaccines

Conjugate capsular vaccines, in which a polysaccharide of interest is conjugated onto a protein carrier, have been shown to be highly successful in preventing infection by *H. influenzae* type b (Hib) (Murphy et al., 1993), *N. meningitidis* (Martinon-Torres et al., 2021) and *Streptococcus pneumoniae* (Kellner et al., 2008). The introduction of the Hib capsular vaccine in the late 1980s led to a dramatic reduction in Hib infections, particularly in high-income countries (Peltola, 2000), that remained low for nearly a decade without a substantial increase of infection by other capsular types. This led to the impression that replacement by other capsule types would not be a problem, which is in stark contrast to the subsequent experience with capsular vaccines for *S. pneumoniae* and *N. meningitidis*. An early study demonstrating capsule switching between group B and C in *N. meningitidis* without vaccine-induced selective pressure wisely predicted that capsule switching could have important consequences for conjugate capsular vaccines (Swartley et al., 1997) that have since been realized. The introduction of the *S. pneumoniae* 7-valent conjugate capsular vaccine led to changes in nasopharyngeal colonization (Kellner et al., 2008) and infection with accompanying evidence that a virulent serotype 4 strain had acquired the genes for production of the serotype 19A capsule (Croucher et al., 2011; Golubchik et al., 2012). The production of vaccines against an increasing number of *S. pneumoniae* capsular types clearly illustrate the challenges these vaccines face. The complexities of microbial communities and our limited understanding of how they are affected by vaccines make predictions of outcomes challenging such that even the dramatic impact on epidemics of meningitis in Sub-Sarahan Africa by the MenAfriVac vaccine targeting the group A capsule is facing concerns of replacement by other capsular types (WHO, 2017).

The demonstration of capsule switching without (Swartley et al., 1997) and with (Croucher et al., 2011) vaccine-induced selective pressure also has important implications regarding classical serology used for epidemiological purposes that often are used to decide on commercial vaccine compositions. A common approach for preventing diseases caused by animal pathogens is the widespread use of inactivated whole-cell vaccines (bacterins) (Liu et al., 2016). As with conjugate capsular vaccines, the protection induced by inactivated whole-cell vaccines is largely attributed to being directed against the extracellular capsular polysaccharide. This results in a highly specific protective response that is unable to yield any cross-protection against strains expressing other capsular types (Takahashi et al., 2001; Bak and Riising, 2002).

An early study of selected strains representing 15 serovars of *G. parasuis* that classified them for their virulence potential (Kielstein and Rapp-Gabrielson, 1992; Oliveira and Pijoan, 2004) has been used to justify the inclusion of strains expressing specific capsular types into a bacterin formulation. Strains belonging to serovar 7 were considered to have low virulence potential since the *G. parasuis* serovar 7 reference strain 174 was unable to cause disease in piglets (Kielstein and Rapp-Gabrielson, 1992; Brockmeier et al., 2014). However, outbreaks of Glässer’s disease with serovar 7 strains in swine herds in China (Wang et al., 2017) and Brazil (Prigol et al., 2022) have since been recorded. Furthermore, experimentally challenging pigs with *G. parasuis* serovar 7 reference strain 174 has been shown to result in the classic symptoms of Glässer’s disease (Frandoloso et al., 2015; Guizzo et al., 2018; Frandoloso et al., 2020; Dazzi et al., 2020). Notably, strains can lose virulence upon passage *in vitro*, which can be restored upon passage in the host, as was implemented in two of these studies (Frandoloso et al., 2020; Dazzi et al., 2020). A recent epidemiological survey of *G. parasuis* serovars in different regions in Brazil demonstrated that most of the circulating strains were not covered by commercially available vaccines, which the authors estimated could have perhaps prevented 28% of the outbreaks reported in that study (Espindola et al., 2019). Notably, there was substantial proportion of ‘non-typeable’ strains that were shown by PCR analysis to have distinct patterns from the known capsular serotypes.

In contrast to bacterins and conjugate capsular vaccines that do not induce a cross-protective immune response, protein-based vaccines have the potential to induce a broad cross-protective immune response, particularly if several protein antigens are used (Rokbi et al., 1997). The distribution of variant TbpBs amongst three porcine pathogens provides the opportunity to prevent infection by all three pathogens with a single multicomponent vaccine (Curran et al., 2015), which might be enhanced further by redesigning the antigens.

## Discussion

Although conjugate capsular vaccines and bacterins that primarily induce an immune response against the capsular polysaccharide have played a very important role in the prevention of bacterial infections in humans and food production animals, there is an ongoing threat of disease arising from bacteria displaying a different capsular type that can be acquired *via* horizontal gene transfer. The potential for replacement with different variants of protein antigens also exists, but the repertoire of variants that can be acquired is restricted by the cross-reactivity of the immune response induced by protein antigens and the specificity of the functions that protein antigens possess. Studies with the porcine ‘pathogens’ *G. parasuis*, *A. pleuropneumoniae*, and *A. suis* have illustrated that even with a limited collection of somewhat geographically diverse isolates one can obtain a reasonable representation of the overall global diversity of the antigens (Curran et al., 2015). The observed distribution of diversity between these three species and the different geographical regions has been maintained with the expanding availability of sequences from public databases and strain collections, but the evolutionary significance of these findings could be strengthened with samples from natural non-pathogenic commensal populations. The potential to eliminate these three species of bacteria from commercial pig barns with a broadly cross-protective vaccine would almost certainly be viewed as worth pursuing even if they may vary in the degree to which they are considered pathogens. Ideally this would be monitored and studied as it could provide insights for human vaccine development.

In the more intensely studied human infections caused by *Neisseria* and *Haemophilus* species, the transferrin receptors have been shown to be present in some of the commensal species, and early studies in *Neisseria* (Harrison et al., 2008) indicated that although some clustering can be observed, it would be challenging to target the immune response exclusively on TbpBs in the pathogenic species. However, more recent analysis indicates that the sequence diversity among gonococcal strains is more restricted, so that a TbpB-based gonococcal vaccine would have a limited impact on ‘commensal’ *Neisseria* species. A TbpB-based vaccine against *H. influenzae* would have the additional advantage of also targeting bacteria that lack a polysaccharide capsule, including the non-typeable *H. influenzae* (NTHi) (Novotny et al., 2017; De Smedt et al., 2021), that cause otitis media in children and exacerbations of chronic obstructive pulmonary disease (COPD) in adults (Murphy, 2015). It would also address the issue of the increasing incidence of meningitis caused by other capsular types of *H. influenzae* (Ulanova and Tsang, 2009). TbpB-based vaccines could also target *Moraxella catarrhalis*, thus potentially extending protection to two of the main causes of otitis media and COPD. A vaccine containing USPA2, a protective *M. catarrhalis* antigen (Ysebaert et al., 2021), and a Protein E-PilA fusion protein from non-typeable *H. influenzae* that protects colonization in mice and Chinchillas (Ysebaert et al., 2019), is already in development but the cross-protective properties of these antigens have not been fully evaluated.

The cross-reactivity of the immune response against the serogroup B polysaccharide capsule of *N. meningitidis* against brain components (Finne et al., 1983) led to early efforts at developing a vaccine targeting TbpB (Rokbi et al., 1997), followed by development of the Trumenba vaccine, which contains two distinct variants of the meningococcal factor H binding protein (fHbp) (Wang et al., 2011), and the Bexsero vaccine, consisting of three surface-exposed protein antigens (NHBA, NadA, fHbp) with outer membrane vesicles (Pizza et al., 2020). The degree to which these vaccines provide comprehensive protection against potential disease isolates and their impact on the commensal species are a function of the antigenic components they target, which provides an opportunity for potentially comparing their potential long-term efficacy.

The Trumemba vaccine, which contains two lipidated factor H binding protein (fHbp) variants, is predicted to provide broad cross-protection from infection (Wang et al., 2011) and the absence of fHbp in *N. lactamica* (Chan et al., 2018) indicates that it would not interfere with the natural protection it provides (Dale et al., 2022). However, the property of binding factor H is not unique to FhBP, strains lacking FhBP are still capable of inhibiting killing by the alternate complement pathway and invasive disease is possible without the ability to bind factor H (Lewis et al., 2013). However, post-licensure analysis of clinical disease isolates for loss or change of factor H binding components can address these concerns. The Bexsero vaccine (Pizza et al., 2020) consists of three antigens selected by reverse vaccinology, two with fusion partners, and includes a detergent extracted outer membrane preparation (OMP) clearly added to increase confidence in coverage. The OMP is responsible for the strong local reaction to immunization with this vaccine. In spite of the complexity of the vaccine composition, an elegant study using a humanized mouse model of colonization with antigenically diverse strains indicates the components of Bexsero induce a protective immune response against sepsis but do not affect nasal carriage (Buckwalter et al., 2017). This reflects vaccine studies in humans (McMillan et al., 2021). The complexity of the vaccine makes post-licensure analysis of efficacy complicated and addressing its impact on the commensal bacteria even more challenging.

The demonstrated importance of the *Neisseria* transferrin receptors for survival on the mucosal surface in humans (Cornelissen et al., 1998) and the *A. pleuropneumoniae* transferrin receptors pigs (Baltes et al., 2002) makes them ideal targets for a vaccine. The demonstrated efficacy of protection induced by mutant TbpBs against infection (Frandoloso et al., 2015) and colonization (Frandoloso et al., 2020) in their native host provides proof of concept for their protective efficacy in humans. The presence of TbpBs in commensal *Neisseria* species does raise potential concern regarding the impact of TbpB-based vaccines on the microbiome, but whether bacteria not intimately associated with the epithelial surface (**Figure 2**) will be as susceptible to adaptive immune mechanisms is an important question. One advantage that TbpB-based vaccines provide is the potential ability to implement studies in other hosts to address the key issues prior to introduction of the vaccine into humans. Tf receptors, which are essential for the survival, proliferation, and pathogenesis of bacteria such as *N. meningitidis* and *H. influenzae* in the human host, have the potential to be superior vaccines due to the greatly diminished likelihood of gene loss due to selection pressure exerted by the host immune response compared to the “non-essential” surface antigens targeted by the Trumenba and Bexsero vaccines.

The hypothesis that there are transferrin and lactoferrin receptors in several bacterial lineages in all mammalian hosts could potentially be addressed by genomic sequences that accumulate over the years from diverse studies. However, specifically addressing the question in rodent species, and particularly wild mice, could potentially provide invaluable tools for enhancing our understanding of the host-commensal-pathogen relationship. Clearly, the initial isolation and identification of Gram-negative bacteria expressing transferrin or lactoferrin receptors that normally reside in the nasal or upper respiratory tract of wild mice may present challenges, but if successful, could lead to considerable insights. The growing spectrum of tools and approaches available in mice combined with approaches for evaluating the impact of the microbiota (McCoy et al., 2019; Kwon and Seong, 2021) could lead to observations that apply broadly to the range of human and animal pathogens that possess transferrin and lactoferrin receptors, including the most effective strategies for inducing an immune response for prevention of infection. Although it may be challenging to secure funding from conventional sources, the ability to perform experiments in parallel in food production animals could provide ‘proof of concept’ that findings in mouse infection models could help predict outcome in humans.

The demonstration that an optimal challenge dose in an intranasal challenge model for *G. parasuis* infection in pigs provides a more natural route of infection (Frandoloso et al., 2020) may apply to other infections, but does not fully replicate the natural route of infection. Optimizing the ‘seeder-pig challenge’ model (Lechtenberg et al., 1994) would provide the most authentic route of infection, but the degree to which it could be applied to other animal species, including mice, is an open question. Nevertheless, it should be recognized that certain vaccine components that are deemed necessary for regulatory approval of certain vaccines due to the use of non-physiological infection models, may not be required for vaccines that are effective for disease reduction or elimination in the real-world situation.

## Data availability statement

The original contributions presented in the study are included in the article/supplementary material. Further inquiries can be directed to the corresponding author.

## Author contributions

AS conceived, initiated and coordinated writing of the manuscript. NE made significant contributions to the biogeography section and prepared **Figure 2**. SC made significant contribution to the bacterin-conjugate capsular vaccine section. All authors contributed to the article and approved the submitted version.

## References

[B1] Anderson J. E. Hobbs M. M. Biswas G. D. Sparling P. F. (2003). Opposing selective forces for expression of the gonococcal lactoferrin receptor. Mol. Microbiol. 48 (5), 1325–1337. doi: 10.1046/j.1365-2958.2003.03496.x 12787359

[B2] Atto B. Kunde D. Gell D. A. Tristram S. (2021). Haemophilin-producing strains of haemophilus haemolyticus protect respiratory epithelia from NTHi colonisation and internalisation. Pathogens 10 (1), 1-15. doi: 10.3390/pathogens10010029 PMC7823694 33401487

[B3] Azimi S. Lewin G. R. Whiteley M. (2022). The biogeography of infection revisited. Nat. Rev. Microbiol. 20, 579–592. doi: 10.1038/s41579-022-00683-3 PMC9357866 35136217

[B4] Bak H. Riising H. J. (2002). Protection of vaccinated pigs against experimental infections with homologous and heterologous haemophilus parasuis. Vet. Rec 151 (17), 502–505. doi: 10.1136/vr.151.17.502 12430998

[B5] Baltes N. Hennig-Pauka I. Gerlach G. F. (2002). Both transferrin binding proteins are virulence factors in *Actinobacillus pleuropneumoniae* serotype 7 infection. FEMS Microbiol. Lett. 209 (2), 283–287. doi: 10.1111/j.1574-6968.2002.tb11145.x 12007819

[B6] Barber M. F. Elde N. C. (2014). Nutritional immunity. escape from bacterial iron piracy through rapid evolution of transferrin. Science 346 (6215), 1362–1366. doi: 10.1126/science.1259329 25504720 PMC4455941

[B7] Brockmeier S. L. Register K. B. Kuehn J. S. Nicholson T. L. Loving C. L. Bayles D. O.. (2014). Virulence and draft genome sequence overview of multiple strains of the swine pathogen haemophilus parasuis. PloS One 9 (8), e103787. doi: 10.1371/journal.pone.0103787 25137096 PMC4138102

[B8] Buckwalter C. M. Currie E. G. Tsang R. S. W. Gray-Owen S. D. (2017). Discordant effects of licensed meningococcal serogroup b vaccination on invasive disease and nasal colonization in a humanized mouse model. J. Infect. Dis. 215 (10), 1590–1598. doi: 10.1093/infdis/jix162 28368526 PMC5461428

[B9] Calmettes C. Yu R.-H. Silva L. P. Curran D. Schriemer D. C. Schryvers A. B.. (2011). Structural variations within the transferrin binding site on transferrin binding protein, TbpB. J. Biol. Chem. 286, 12683–12692. doi: 10.1074/jbc.M110.206102 21297163 PMC3069468

[B10] Chan C. Andisi V. F. Ng D. Ostan N. Yunker W. K. Schryvers A. B. (2018). Are lactoferrin receptors in gram-negative bacteria viable vaccine targets? Biometals 31 (3), 381–398. doi: 10.1007/s10534-018-0105-7 29767396

[B11] Conlon J. A. Shewen P. E. Lo R. Y. C. (1991). Efficacy of recombinant leukotoxin in protection against pneumonic challenge with live pasteurella haemolytica A1. Infect Immun. 59, 587–591. doi: 10.1128/iai.59.2.587-591.1991 1987075 PMC257793

[B12] Cornelissen C. N. Kelley M. Hobbs M. M. Anderson J. E. Cannon J. G. Cohen M. S.. (1998). The transferrin receptor expressed by gonococcal strain FA1090 is required for the experimental infection of human male volunteers. Mol. Microbiol. 27 (3), 611–616. doi: 10.1046/j.1365-2958.1998.00710.x 9489672

[B13] Crepin V. F. Collins J. W. Habibzay M. Frankel G. (2016). Citrobacter rodentium mouse model of bacterial infection. Nat. Protoc. 11 (10), 1851–1876. doi: 10.1038/nprot.2016.100 27606775

[B14] Croucher N. J. Harris S. R. Fraser C. Quail M. A. Burton J. van der Linden M.. (2011). Rapid pneumococcal evolution in response to clinical interventions. Science 331 (6016), 430–434. doi: 10.1126/science.1198545 21273480 PMC3648787

[B15] Curran D. Adamiak P. Fegan J. Qian C. Yu R. Schryvers A. B. (2015). Sequence and structural diversity of transferrin receptors in gram-negative porcine pathogens. Vaccine 33 (42), 5700–5707. doi: 10.1016/j.vaccine.2015.07.097 26263196

[B16] D'Onofrio A. Crawford J. M. Stewart E. J. Witt K. Gavrish E. Epstein S.. (2010). Siderophores from neighboring organisms promote the growth of uncultured bacteria. Chem. Biol. 17 (3), 254–264. doi: 10.1016/j.chembiol.2010.02.010 20338517 PMC2895992

[B17] Dale A. P. Theodosiou A. A. Gbesemete D. F. Guy J. M. Jones E. F. Hill A. R.. (2022). Effect of colonisation with neisseria lactamica on cross-reactive anti-meningococcal b-cell responses: a randomised, controlled, human infection trial. Lancet Microbe 3 (12), e931–e43. doi: 10.1016/S2666-5247(22)00283-X 36462524 PMC7615047

[B18] Danve B. Lissolo L. Mignon M. Dumas P. Colombani S. Schryvers A. B.. (1993). Transferrin-binding proteins isolated from *Neisseria meningitidis* elicit protective and bactericidal antibodies in laboratory animals. Vaccine 11, 1214–1220. doi: 10.1016/0264-410X(93)90045-Y 8256502

[B19] Dazzi C. C. Guizzo J. A. Prigol S. R. Kreutz L. C. Driemeier D. Chaudhuri S.. (2020). New pathological lesions developed in pigs by a "Non-virulent" strain of glaesserella parasuis. Front. Vet. Sci. 7, 98. doi: 10.3389/fvets.2020.00098 32158772 PMC7052124

[B20] De Smedt P. Leroux-Roels G. Vandermeulen C. Tasciotti A. Di Maro G. Dozot M.. (2021). Long-term immunogenicity and safety of a non-typeable haemophilus influenzae-moraxella catarrhalis vaccine: 4-year follow-up of a phase 1 multicentre trial. Vaccine X. 9, 100124. doi: 10.1016/j.jvacx.2021.100124 34820619 PMC8600057

[B21] Diallo K. MacLennan J. Harrison O. B. Msefula C. Sow S. O. Daugla D. M.. (2019). Genomic characterization of novel neisseria species. Sci. Rep. 9 (1), 13742. doi: 1038/s41598-019-50203-2 31551478 10.1038/s41598-019-50203-2 PMC6760525

[B22] Ekins A. Bahrami F. Sijercic A. Maret D. Niven D. F. (2004). Haemophilus somnus possesses two systems for acquisition of transferrin-bound iron. J. Bacteriol. 186 (13), 4407–4411. doi: 10.1128/JB.186.13.4407-4411.2004 15205447 PMC421612

[B23] Espindola J. P. Balbinltt N. Gressler L. T. Machado G. Klein C. S. Rebelatto R.. (2019). Molecular serotyping of clinical strains of haemophilus (Glasserella) parasuis brings new insights regarding glasser’s disease outbreaks in Brazil. PeerJ 7, e6817. doi: 10.7717/peerj.6817 31198621 PMC6535215

[B24] Fegan J. E. Calmettes C. Islam E. A. Ahn S. K. Chaudhuri S. Yu R. H.. (2019). Utility of hybrid transferrin binding protein antigens for protection against pathogenic neisseria species. Front. Immunol. 10, 247. doi: 10.3389/fimmu.2019.00247 30837995 PMC6389628

[B25] Finne J. Leinonen M. Makela P. H. (1983). Antigenic similarities between brain components and bacteria causing meningitis. Lancet ii, 355–357. doi: 10.1016/S0140-6736(83)90340-9 6135869

[B26] Frandoloso R. Chaudhuri S. Frandoloso G. C. P. Yu R. H. Schryvers A. B. (2020). Proof of concept for prevention of natural colonization by oral needle-free administration of a microparticle vaccine. Front. Immunol. 11, 595320. doi: 10.3389/fimmu.2020.595320 33193449 PMC7645216

[B27] Frandoloso R. Martinez-Martinez S. Calmettes C. Fegan J. Costa E. Curran D.. (2015). Nonbinding site-directed mutants of transferrin binding protein b enhances their immunogenicity and protective capabilities. Infect. Immun. 83 (3), 1030–1038. doi: 10.1128/IAI.02572-14 25547790 PMC4333475

[B28] Golubchik T. Brueggemann A. B. Street T. Gertz R. E. Jr. Spencer C. C. Ho T.. (2012). Pneumococcal genome sequencing tracks a vaccine escape variant formed through a multi-fragment recombination event. Nat. Genet. 44 (3), 352–355. doi: 10.1038/ng.1072 22286217 PMC3303117

[B29] Gonzalez G. C. Caamano D. L. Schryvers A. B. (1990). Identification and characterization of a porcine-specific transferrin receptor in actinobacillus pleuropneumoniae. Mol. Microbiol. 4, 1173–1179. doi: 10.1111/j.1365-2958.1990.tb00692.x 2233254

[B30] Gray-Owen S. D. Blumberg R. S. (2006). CEACAM 1: contact-dependent control of immunity. Nat. Reviews/Immunol 6 (11), 433–446. doi: 10.1038/nri1864 16724098

[B31] Gray-Owen S. D. Schryvers A. B. (1993). The interaction of primate transferrins with receptors on bacteria pathogenic to humans. Microbial Pathogen 14, 389–398. doi: 10.1006/mpat.1993.1038 8366816

[B32] Gray-Owen S. D. Schryvers A. B. (1996). Bacterial transferrin and lactoferrin receptors. Trends Microbiol. 4 (5), 185–191. doi: 10.1016/0966-842X(96)10025-1 8727598

[B33] Guizzo J. A. Chaudhuri S. Prigol S. R. Yu R. H. Dazzi C. C. Balbinott N.. (2018). The amino acid selected for generating mutant TbpB antigens defective in binding transferrin can compromise the *in vivo* protective capacity. Sci. Rep. 8 (1), 7372. doi: 10.1038/s41598-018-25685-1 29743502 PMC5943581

[B34] Harrison O. B. Maiden M. C. Rokbi B. (2008). Distribution of transferrin binding protein b gene (tbpB) variants among neisseria species. BMC Microbiol. 8, 66. doi: 10.1186/1471-2180-8-66 18430216 PMC2386816

[B35] Johswich K. O. McCaw S. E. Islam E. Sintsova A. Gu A. Shively J. E.. (2013). *In vivo* adaptation and persistence of neisseria meningitidis within the nasopharyngeal mucosa. PloS Pathog. 9 (7), e1003509. doi: 10.1371/journal.ppat.1003509 23935487 PMC3723569

[B36] Kellner J. Scheifele D. Vanderkooi O. MacDonald J. Church D. (2008). Effects of routine infant vaccination with the 7-valent pneumococcal conjugate vaccine on nasopharyngeal colonization with streptococcus pneumoniae in children in Calgary, Canada. Pediatr. Infect. Dis. J. 27, 526–532. doi: 10.1097/INF.0b013e3181658c5c 18458650

[B37] Kielstein P. Rapp-Gabrielson V. J. (1992). Designation of 15 serovars of haemophilus parasuis on the basis of immunodiffusion using heat-stable antigen extracts. J. Clin. Microbiol. 30 (4), 862–865. doi: 10.1128/jcm.30.4.862-865.1992 1572971 PMC265175

[B38] Kwon H. K. Seong J. K. (2021). New insights into the microbiota of wild mice. Mamm Genome 32 (4), 311–318. doi: 10.1007/s00335-021-09887-z 34241667 PMC8295133

[B39] Lambert L. A. (2012). Molecular evolution of the transferrin family and associated receptors. Biochim. Biophys. Acta 1820 (3), 244–255. doi: 10.1016/j.bbagen.2011.06.002 21693173

[B40] Lambert L. A. Perri H. Meehan T. J. (2005). Evolution of duplications in the transferrin family of proteins. Comp. Biochem. Physiol. B Biochem. Mol. Biol. 140 (1), 11–25. doi: 10.1016/j.cbpc.2004.09.012 15621505

[B41] Large R. R. Hazen R. M. Morrison S. M. Gregory D. D. Steadman J. A. Mukherjee I. (2022). Evidence that the GOE was a prolonged event with a peak around 1900 ma. Geosyst Geoenviron 1 (2), 100036. doi: 10.1016/j.geogeo.2022.100036

[B42] Lechtenberg K. F. Shryock T. R. Moore G. (1994). Characterization of an actinobacillus pleuropneumoniae seeder pig challenge-exposure model. Am. J. Vet Res. 55, 1703–1709. doi: doi.org/10.1128/IAI.00133-06 7887514

[B43] Lewis L. A. Vu D. M. Vasudhev S. Shaughnessy J. Granoff D. M. Ram S. (2013). Factor h-dependent alternative pathway inhibition mediated by porin b contributes to virulence of neisseria meningitidis. mBio 4 (5), e00339–e00313. doi: 10.1128/mBio.00339-13 24129254 PMC3812710

[B44] Lissolo L. Maitre-Wilmotte G. Dumas P. Mignon M. Danve B. Quentin-Millet M.-J. (1995). Evaluation of transferrin-binding protein 2 within the transferrin- binding protein complex as a potential antigen for future meningococcal vaccines. Infect Immun. 63 (3), 884–890. doi: 10.1128/iai.63.3.884-890.1995 7868259 PMC173085

[B45] Liu H. Xue Q. Zeng Q. Zhao Z. (2016). Haemophilus parasuis vaccines. Vet. Immunol. Immunopathol. 180, 53–58. doi: 10.1016/j.vetimm.2016.09.002 27692096

[B46] Ma M. Powell D. A. Weyand N. J. Rhodes K. A. Rendon M. A. Frelinger J. A.. (2018). A natural mouse model for neisseria colonization. Infect. Immun. 86 (5). doi: 10.1128/IAI.00839-17 PMC5913851 29440372

[B47] Martinon-Torres F. Banzhoff A. Azzari C. De Wals P. Marlow R. Marshall H.. (2021). Recent advances in meningococcal b disease prevention: real-world evidence from 4CMenB vaccination. J. Infect. 83 (1), 17–26. doi: 10.1016/j.jinf.2021.04.031 33933528

[B48] McCoy K. D. Burkhard R. Geuking M. B. (2019). The microbiome and immune memory formation. Immunol. Cell Biol. 97 (7), 625–635. doi: 10.1111/imcb.12273 31127637

[B49] McMillan M. Chandrakumar A. Wang H. L. R. Clarke M. Sullivan T. R. Andrews R. M.. (2021). Effectiveness of meningococcal vaccines at reducing invasive meningococcal disease and pharyngeal neisseria meningitidis carriage: A systematic review and meta-analysis. Clin. Infect. Dis. 73 (3), e609–ee19. doi: 10.1093/cid/ciaa1733 33212510

[B50] Moraes T. F. Yu R.-H. Strynadka N. C. Schryvers A. B. (2009). Insights into the bacterial transferrin receptor: the structure of transferrin binding protein b from actinobacillus pleuropneumoniae. Mol. Cell 35 (4), 523–533. doi: 10.1016/j.molcel.2009.06.029 19716795

[B51] Murphy T. F. (2015). Vaccines for nontypeable haemophilus influenzae: the future is now. Clin. Vaccine Immunol. 22 (5), 459–466. doi: 10.1128/CVI.00089-15 25787137 PMC4412935

[B52] Murphy T. V. White K. E. Pastor P. Gabriel L. Medley F. Granoff D. M.. (1993). Declining incidence of haemophilus influenzae type b disease since introduction of vaccination. Jama 269 (2), 246–248. doi: 10.1001/jama.1993.03500020080036 8417244

[B53] Noinaj N. Easley N. C. Oke M. Mizuno N. Gumbart J. Boura E.. (2012). Structural basis for iron piracy by pathogenic neisseria. Nature 483, 53–58. doi: 10.1038/nature10823 22327295 PMC3292680

[B54] Novotny L. A. Clements J. D. Goodman S. D. Bakaletz L. O. (2017). Transcutaneous immunization with a band-aid prevents experimental otitis media in a polymicrobial model. Clin. Vaccine Immunol. 24 (6), 1–12. doi: 10.1128/CVI.00563-16 PMC5461379 28381402

[B55] Ogunnariwo J. A. Cheng C. Y. Ford J. A. Schryvers A. B. (1990). Response of *Haemophilus somnus* to iron limitation: Expression and identification of a bovine-specific transferrin receptor. Microbial Pathogen 9, 397–406. doi: 10.1016/0882-4010(90)90058-X 2151461

[B56] Ogunnariwo J. A. Schryvers A. B. (1990). Iron acquisition in *Pasteurella haemolytica*: Expression and identification of a bovine-specific transferrin receptor. Infect Immun. 58, 2091–2097. doi: 10.1128/iai.58.7.2091-2097.1990 2365453 PMC258781

[B57] Ogunnariwo J. A. Schryvers A. B. (1992). Correlation between the ability of *Haemophilus paragallinarum* to acquire ovotransferrin-bound iron and the expression of ovotransferrin- specific receptors. Avian Dis. 36, 655–663. doi: 10.2307/1591761 1417595

[B58] Ogunnariwo J. A. Schryvers A. B. (2001). Characterization of a novel transferrin receptor in bovine strains of pasteurella multocida. J. Bacteriol 183 (3), 890–896. doi: 10.1128/JB.183.3.890-896.2001 11208786 PMC94955

[B59] Oliveira S. Pijoan C. (2004). Haemophilus parasuis: New trends on diagnosis, epidemiology and control. Vet. Microbiol. 99 (1), 1–12. doi: 10.1016/j.vetmic.2003.12.001 15019107

[B60] Ostan N. K. H. Moraes T. F. Schryvers A. B. (2021). Lactoferrin receptors in gram-negative bacteria: an evolutionary perspective. Biochem. Cell Biol. 99 (1), 102–108. doi: 10.1139/bcb-2020-0079 33464172

[B61] Peltola H. (2000). Worldwide haemophilus influenzae type b disease at the beginning of the 21st century: global analysis of the disease burden 25 years after the use of the polysaccharide vaccine and a decade after the advent of conjugates. Clin. Microbiol. Rev. 13 (2), 302–317. doi: 10.1128/CMR.13.2.302 10756001 PMC100154

[B62] Pizza M. Bekkat-Berkani R. Rappuoli R. (2020). Vaccines against meningococcal diseases. Microorganisms 8 (1521), 1–21. doi: 10.3390/microorganisms8101521 PMC7601370 33022961

[B63] Postle K. (1993). TonB protein and energy transduction between membranes. JBioenergBiomembr 25, 591–601. doi: 10.1007/BF00770246 8144488

[B64] Prigol S. R. Klein R. Chaudhuri S. Frandoloso G. P. Guizzo J. A. Gutierrez Martin C.. (2022). TbpBY167A-based vaccine can protect pigs against glässer’s disease triggered by glaesserella parasuis SV7 expressing TbpB cluster I. Pathogens 11 (766), 1–14. doi: 10.3390/pathogens11070766 PMC9323293 35890011

[B65] Rokbi B. Mignon M. Maitre-Wilmotte G. Lissolo L. Danve B. Caugant D. A.. (1997). Evaluation of recombinant transferrin binding protein b variants from *Neisseria meningitidis* for their ability of induce cross reactive and bactericidal antibodies against a genetically diverse collection of serogroup b strains. Infect Immun. 65 (1), 55–63. doi: 10.1128/iai.65.1.55-63.1997 8975892 PMC174556

[B66] Schryvers A. B. Morris L. J. (1988a). Identification and characterization of the transferrin receptor from neisseria meningitidis. Mol. Microbiol. 2, 281–288. doi: 10.1111/j.1365-2958.1988.tb00029.x 3132585

[B67] Schryvers A. B. Morris L. J. (1988b). Identification and characterization of the human lactoferrin-binding protein from neisseria meningitidis. Infect Immun. 56, 1144–1149. doi: 10.1128/iai.56.5.1144-1149.1988 3128478 PMC259775

[B68] Singh B. Al-Jubair T. Morgelin M. Thunnissen M. M. Riesbeck K. (2013). The unique structure of haemophilus influenzae protein e reveals multiple binding sites for host factors. Infect. Immun. 81 (3), 801–814. doi: 10.1128/IAI.01111-12 23275089 PMC3584867

[B69] Sosa Torres M. E. Saucedo-Vázquez J. P. Kroneck P. M. (2015). The magic of dioxygen. Met Ions Life Sci. 15, 1–12. doi: 10.1007/978-3-319-12415-5_1 25707464

[B70] Swanson M. T. Oliveros C. H. Esselstyn J. A. (2019). A phylogenomic rodent tree reveals the repeated evolution of masseter architectures. Proc. Biol. Sci. 286 (1902), 20190672. doi: 10.1098/rspb.2019.0672 31064307 PMC6532498

[B71] Swartley J. S. Marfin A. A. Edupuganti S. Liu L. J. Cieslak P. Perkins B.. (1997). Capsule switching of neisseria meningitidis. Proc. Natl. Acad. Sci. U.S.A. 94 (1), 271–276. doi: 10.1073/pnas.94.1.271 8990198 PMC19312

[B72] Takahashi K. Naga S. Yagihashi T. Ikehata T. Nakano Y. Senna K.. (2001). A cross-protection experiment in pigs vaccinated with haemophilus parasuis serovars 2 and 5 bacterins, and evaluation of a bivalent vaccine under laboratory and field conditions. J. Vet. Med. Sci. 63 (5), 487–491. doi: 10.1292/jvms.63.487 11411491

[B73] Tatum F. M. Briggs R. E. Sreevatsan S. S. Zehr E. S. Hsuan S. L. Whiteley L. O.. (1998). Construction of an isogenic leukotoxin deletion mutant of pasteurella haemolytica serotype 1: characterization and virulence. Microbial Pathogen 24 (1), 37–46. doi: 10.1006/mpat.1997.0181 9466945

[B74] Ulanova M. Tsang R. S. (2009). Invasive haemophilus influenzae disease: changing epidemiology and host-parasite interactions in the 21st century. Infect. Genet. Evol. 9 (4), 594–605. doi: 10.1016/j.meegid.2009.03.001 19460326

[B75] Wanford J. J. Green L. R. Aidley J. Bayliss C. D. (2018). Phasome analysis of pathogenic and commensal neisseria species expands the known repertoire of phase variable genes, and highlights common adaptive strategies. PloS One 13 (5), e0196675. doi: 10.1371/journal.pone.0196675 29763438 PMC5953494

[B76] Wang X. Cohn A. Comanducci M. Andrew L. Zhao X. MacNeil J. R.. (2011). Prevalence and genetic diversity of candidate vaccine antigens among invasive neisseria meningitidis isolates in the united states. Vaccine 29 (29-30), 4739–4744. doi: 10.1016/j.vaccine.2011.04.092 21571026

[B77] Wang Z. Zhao Q. Wei H. Wen X. Cao S. Huang X.. (2017). Prevalence and seroepidemiology of haemophilus parasuis in sichuan province, China. PeerJ 5, e3379. doi: 10.7287/peerj.preprints.2805v1 28584712 PMC5452937

[B78] Weyand N. J. Ma M. Phifer-Rixey M. Taku N. A. Rendon M. A. Hockenberry A. M.. (2016). Isolation and characterization of neisseria musculi sp. nov., from the wild house mouse. Int. J. Syst. Evol. Microbiol. 66 (9), 3585–3593. doi: 10.1099/ijsem.0.001237 27298306 PMC5880574

[B79] WHO (2017). Serogroup c in the meningitis belt: What is next? (Geneva, Switzerland: World Health Organization), 1–10.

[B80] Yang X. Yu R. H. Calmettes C. Moraes T. F. Schryvers A. B. (2011). The anchor peptide of transferrin binding protein b is required for interaction with transferrin binding protein a. J. Biol. Chem. 286 (52), 45165–45173. doi: 10.1074/jbc.M110.214171 22069313 PMC3247978

[B81] Ysebaert C. Castado C. Mortier M. C. Rioux S. Feron C. Di Paolo E.. (2021). UspA2 is a cross-protective moraxella catarrhalis vaccine antigen. Vaccine 39 (39), 5641–5649. doi: 10.1016/j.vaccine.2021.08.002 34446318

[B82] Ysebaert C. Denoel P. Weynants V. Bakaletz L. O. Novotny L. A. Godfroid F.. (2019). A protein e-PilA fusion protein shows vaccine potential against nontypeable haemophilus influenzae in mice and chinchillas. Infect. Immun. 87 (8), 1–13. doi: 10.1128/IAI.00345-19 PMC6652774 31109946

